# Whole Grain, Bran, and Germ Intake and Risk of Type 2 Diabetes: A Prospective Cohort Study and Systematic Review

**DOI:** 10.1371/journal.pmed.0040261

**Published:** 2007-08-28

**Authors:** Jeroen S. L de Munter, Frank B Hu, Donna Spiegelman, Mary Franz, Rob M van Dam

**Affiliations:** 1 Department of Nutrition, Harvard School of Public Health, Boston, Massachusetts, United States of America; 2 Institute of Health Sciences, Vrije Universiteit Amsterdam, Amsterdam, The Netherlands; 3 Department of Epidemiology, Harvard School of Public Health, Boston, Massachusetts, United States of America; 4 Channing Laboratory, Department of Medicine, Brigham and Women's Hospital and Harvard Medical School; 5 Department of Biostatistics, Harvard School of Public Health, Boston, Massachusetts, United States of America; Clinical Research Centre, Sweden

## Abstract

**Background:**

Control of body weight by balancing energy intake and energy expenditure is of major importance for the prevention of type 2 diabetes, but the role of specific dietary factors in the etiology of type 2 diabetes is less well established. We evaluated intakes of whole grain, bran, and germ in relation to risk of type 2 diabetes in prospective cohort studies.

**Methods and Findings:**

We followed 161,737 US women of the Nurses' Health Studies (NHSs) I and II, without history of diabetes, cardiovascular disease, or cancer at baseline. The age at baseline was 37–65 y for NHSI and 26–46 y for NHSII. Dietary intakes and potential confounders were assessed with regularly administered questionnaires. We documented 6,486 cases of type 2 diabetes during 12–18 y of follow-up. Other prospective cohort studies on whole grain intake and risk of type 2 diabetes were identified in searches of MEDLINE and EMBASE up to January 2007, and data were independently extracted by two reviewers. The median whole grain intake in the lowest and highest quintile of intake was, respectively, 3.7 and 31.2 g/d for NHSI and 6.2 and 39.9 g/d for NHSII. After adjustment for potential confounders, the relative risks (RRs) for the highest as compared with the lowest quintile of whole grain intake was 0.63 (95% confidence interval [CI] 0.57–0.69) for NHSI and 0.68 (95% CI 0.57–0.81) for NHSII (both: *p*-value, test for trend <0.001). After further adjustment for body mass index (BMI), these RRs were 0.75 (95% CI 0.68–0.83; *p*-value, test for trend <0.001) and 0.86 (95% CI 0.72–1.02; *p*-value, test for trend 0.03) respectively. Associations for bran intake were similar to those for total whole grain intake, whereas no significant association was observed for germ intake after adjustment for bran. Based on pooled data for six cohort studies including 286,125 participants and 10,944 cases of type 2 diabetes, a two-serving-per-day increment in whole grain consumption was associated with a 21% (95% CI 13%–28%) decrease in risk of type 2 diabetes after adjustment for potential confounders and BMI.

**Conclusions:**

Whole grain intake is inversely associated with risk of type 2 diabetes, and this association is stronger for bran than for germ. Findings from prospective cohort studies consistently support increasing whole grain consumption for the prevention of type 2 diabetes.

## Abstract 2

Jeroen de Munter and colleagues found that, in women in the US Nurses' Health Studies, whole grain intake was inversely associated with risk of type 2 diabetes. The association was stronger for bran than for germ.

## Editors' Summary

**Background.:**

Type 2 diabetes mellitus (also sometimes called adult-onset or noninsulin-dependent diabetes) is increasing worldwide and is the most common form of diabetes. It puts people at risk of poor health and death by increasing their risk of heart disease and stroke, and a range of other conditions including blindness, kidney disease, and ulcers. It has long been recognized that there is a link between diet and developing type 2 diabetes, because people who are overweight (because the amount of energy in their diet is greater than the energy they use up) run a greater risk of getting type 2 diabetes. However, it has not been clear which particular nutrients or foods might increase the risk or might give protection.

Cereals—such as rice, wheat, corn (maize), etc.—make up a major part of most people's diets. During the refining of cereal grains, much of the outer part of the grain (kernel) are usually removed. Foods are described as “whole grain” if all components of the kernel (the bran, germ, and endosperm) are still present in their natural proportions. There is good evidence that consumption of whole grains may reduce the risk of several diseases, including various types of cancer, heart attacks, and strokes. Some evidence also suggests that eating a diet rich in whole grains might help protect against diabetes, but this has not been firmly established.

**Why Was This Study Done?:**

The authors of this study wanted to find out how much whole grain was eaten by a large number of people over several years and to record how many of these people developed type 2 diabetes. If these two things were closely associated it would provide more evidence to support the idea that whole grain consumption helps protect against type 2 diabetes.

**What Did the Researchers Do and Find?:**

The researchers drew on information recorded in a very large and continuing study in the US, the Nurses' Health Study, which began in 1976, when over 100,000 female registered US nurses completed and returned a mailed questionnaire to assess their health and lifestyle. More nurses were added in 1989. It is an example of what is known as a “cohort study.” Every two years, questionnaires have been mailed to the nurses. Questions asked include the nurses' age, weight, their diet, whether they smoke, their use of oral contraception; and their personal history of diabetes, cardiovascular disease, and cancer. The researchers calculated each nurse's whole grain intake in grams per day. They found that by 2004 about 6,500 of them had developed type 2 diabetes. From an analysis of the data it was clear that the greater the consumption of whole grains the lower the risk of getting type 2 diabetes.

An additional part of the study was that the researchers searched the medical literature for other cohort studies that examined whole grain intake in relation to risk of type 2 diabetes. (This type of research is called a “systematic review,” and it requires that researchers define clearly in advance the kind of studies they are looking for and how they will analyze the data.) They found five such studies. They added together the results of all the studies, including their own. This gave a total of nearly 11,000 cases of type 2 diabetes, out of around 286,000 people. From their analysis they calculated that a two-serving-per-day increment in whole grain consumption was associated with a 21% decrease in risk of type 2 diabetes.

**What Do These Findings Mean?:**

Scientists say that association can never prove causation. (That would require a different sort of study called a trial, where two similar groups of people would be given either a diet high in whole grains or one that was low.) Nevertheless, the research does strongly suggest that a healthy diet that reduces the risk of developing type 2 diabetes should include the consumption of several servings of whole grains daily. The authors do point out that people who choose to eat a lot of whole grains also tend to have a healthy lifestyle in other respects, and that it was hard to calculate intake accurately. However, they do not consider that these limitations to their study would have affected the overall result too seriously.

**Additional Information.:**

Please access these Web sites via the online version of this summary at http://dx.doi.org/10.1371/journal.pmed.0040261.

- Good introductory information about diabetes (type 1 and type 2) may be found on the Web sites of the National Diabetes Clearing House (US) and Diabetes UK
- More detailed information is available on Medline Plus, a Web site that brings together authoritative information from several US government agencies and health-related organizations
- Wikipedia has an entry on whole grain (Wikipedia is a free online encyclopedia that anyone can edit)
- The Nurses' Health Study has a Web site

## Introduction

The prevalence of type 2 diabetes is increasing rapidly worldwide [1]. Control of body weight by balancing energy intake and energy expenditure is of major importance for the prevention of type 2 diabetes, but the role of specific dietary factors in the etiology of type 2 diabetes is less well established [2]. Evidence is accumulating that consumption of whole grains may reduce risk of chronic diseases including various types of cancer [3], cardiovascular diseases [4], and type 2 diabetes [5–9]. Foods are considered to be whole grains if all components of the kernel, i.e., the bran, germ, and endosperm, are present in their natural proportions. Both the fiber-rich bran outer coating and the inner germ are rich in micronutrients and phytochemicals, whereas the endosperm middle layer mainly consists of starch. In the refining process, components of the grain that are part of the bran and germ are lost, including fiber, minerals, vitamins, lignans, and other phytochemicals [10]. These components may offer important health benefits, including beneficial effects on glucose metabolism [11–13].

In most previous studies, foods are defined as whole grains if at least 25% is whole grain or bran by weight [14]. We used a recently developed food composition database of the grams of whole grains per food to directly calculate each participant's whole grain intake in grams per day [15]. This approach avoids the use of an arbitrary cut-point to classify a food as a whole grain food. In addition, our food composition database now includes bran and germ separately; these whole grain constituents have not to our knowledge been studied in relation to risk of type 2 diabetes before. The endosperm is fairly stable at about 80% of the entire grain, but the proportions of bran and germ can vary by cereal type.

We previously reported data on whole grain intake and risk of type 2 diabetes after 10 y of follow-up [5]. Here, we extend this analysis to 18 y of follow-up, include data from the Nurses' Health Study (NHS) II, use the quantitative whole grain variable, and evaluate the role of the bran and germ constituents separately. Furthermore, we combine our results with those from previous cohort studies in a meta-analysis to systematically evaluate the strength of the epidemiological evidence for a relation between whole grain intake and risk of type 2 diabetes.

## Methods

### Study Population

The NHSI began in 1976, when 121,700 female registered US nurses completed and returned a mailed questionnaire. Every 2 y since, questionnaires have been mailed to assess health and lifestyle. Because the 1984 food frequency questionnaire (FFQ) was the first to include a detailed assessment of breakfast cereals, we used 1984, when women were 37–65 y of age, as baseline for the current analysis. The NHSII began in 1989, when 116,609 female registered US nurses completed and returned a mailed questionnaire. Because the first FFQ was administered in 1991, when women were aged 26–46 y, we used that year as baseline for the current analysis. We excluded participants who did not complete the baseline FFQ, left 12 or more (NHSI) or ten or more (NHSII) items blank, or had implausible reported total energy intakes (<600 kcal/d or >3,500 kcal/d). In addition, we excluded participants if they had a history of diabetes (including gestational diabetes), cancer, or cardiovascular disease at baseline (*n* = 7,001 for NHSI and *n* = 6,254 for NHSII), because participants with a diagnosis of these chronic diseases are likely to have changed their diet. For NHSI, the average 1976 (December 31) ages were 42.0 and 42.8 y and average weights were 62.6 and 64.0 kg for the original participants that were included and excluded, respectively. For NHSII, the average 1989 (December 31) ages were 34.0 and 33.8 y, average heights were 165 and 165 cm, and average weights were 65.1 and 66.6 kg for the original participants that were included and excluded, respectively. After exclusions, a total of 73,327 NHSI and 88,410 NHSII participants remained for our present analysis.

### Assessment of Whole Grains

Dietary information was collected using a semiquantitative FFQ that was completed in 1984, 1986, 1990, 1994, and 1998 for NHSI and 1991, 1995, and 1999 for NHSII. The questionnaire asked about average food intake during the past year. Response was given in a commonly used portion size (e.g., a slice of bread) and nine categories of intake ranging from “never, or less than once a month” to “6+ per day”. Open-ended questions were available for breakfast cereal brand names and foods not listed on the FFQ.

The portions were converted to gram weights per serving, and intakes of nutrients were computed by multiplying the frequency of consumption of each unit of food by the nutrient content in grams. Consumption of whole grain (in g/d) was estimated from all grain foods (rice, bread, pasta, and breakfast cereals) based on their dry weight of whole grain ingredients. Whole grain intake from breakfast cereal was derived from more than 250 brand name cereals using information provided by product labels and breakfast cereal manufacturers.

Our whole grain definition included both intact and pulverized forms containing the expected proportion of bran, germ, and endosperm for the specific grain types. The following ingredients in the database were considered whole grains: whole wheat and whole wheat flour, whole oats and whole oat flour, whole cornmeal and whole corn flour, brown rice and brown rice flour, whole rye and whole rye flour, whole barley, bulgur, buckwheat, popcorn, amaranth, and psyllium. Bran and germ in this study refer to total bran and total germ respectively including both the amount naturally contained in whole grains and the amount eaten separately or added during industrial processing or during cooking by the participant.

The method used to develop this whole grain food composition database has been described in detail elsewhere [15]. Our FFQ has been validated extensively using biomarkers and diet records as reference methods [16]. For intakes of cold breakfast cereal and dark bread, major sources of whole grains, the Pearson correlation coefficient for the estimates derived from the FFQ and diet records corrected for within-person variation ranged between 0.58 and 0.79 [17].

### Assessment of Type 2 Diabetes

Cases of diabetes were identified from the mailed questionnaire. Women who reported diabetes were sent an additional questionnaire. Consistent with the criteria of the National Diabetes Data Group [18], diagnosed cases required (1) an elevated glucose concentration (fasting plasma glucose of ≥7.8 mmol/l, random plasma glucose of ≥11.1 mmol/l, or plasma glucose ≥11.1 mmol/l after an oral glucose load), and at least one symptom related to diabetes (excessive thirst, polyuria, weight loss, or hunger); (2) no symptoms, but elevated glucose concentrations on two occasions; and (3) treatment with insulin or oral hypoglycemic medication. For cases of type 2 diabetes identified after 1998, the cut-off point used for fasting plasma glucose concentrations was lowered to 7.0 mmol/l according to the American Diabetes Association criteria [19]. Our validation study showed a high confirmation (98%) of self-reported type 2 diabetes after review of the medical record [20].

### Anthropometry, Medical History, and Lifestyle

Information requested on the baseline questionnaire included age, weight, smoking status, use of postmenopausal hormone therapy, use of oral contraceptives (for NHSII), and personal history of diabetes, cardiovascular disease, and cancer. We updated this information every 2 y. Oral contraceptive use (for NHSI), family history of diabetes, and height were assessed only at baseline. Physical activity data were assessed in 1982, 1986, 1988, 1992, 1996, 1998, and 2000 for NHSI and in 1991 and 1997 for NHSII. Self-administered questionnaires about physical activity and body weight have been validated as described previously [21,22]. We calculated body mass index (BMI) as weight in kilograms divided by the height in meters squared (kg/m^2^).

### Statistical Methods

We used Cox proportional hazards analysis to estimate the relative risk (RR) for type 2 diabetes according to dietary intakes. To control as finely as possible for confounding by age and calendar time, we stratified the analysis jointly by age in months at start of follow-up and calendar year of the current questionnaire cycle. The time scale for the analysis was then measured as months since the start of the current questionnaire cycle. Person-years of follow-up were counted from the date of return from the baseline questionnaire (1984 for NHSI, 1991 for NHSII) until the date of diabetes diagnosis, death, or the end of follow-up (June 2002 for NHSI, June 2003 for NHSII), whichever came first.

Dietary variables were categorized in quintiles of intake. We also conducted analyses modeling whole grain intake as a continuous variable: RR of type 2 diabetes was calculated for a 40 g increment in whole grain intake, which was approximately equivalent to the difference between the 5th and the 95th percentile of intake in our studies (NHSI: 35.9 g, NHSII: 44.3 g). To reduce within-person variation, we used the cumulative average dietary intake from all available dietary questionnaires up to the start of each 2-y follow-up [23]. In NHSII for example, dietary intake reported on the 1991 questionnaire was related to incidence of diabetes from 1991 to 1995, the average of intakes reported on the 1991 and 1995 questionnaires was related to diabetes incidence from 1995 to 1999, and the average of intakes reported on the 1991, 1995, and 1999 questionnaires was related to diabetes incidence from 1999 to 2003.

Nondietary covariates were updated by using the most recently assessed exposure for each 2-y follow-up period. In NHSII for example, smoking status reported on the 1991 questionnaire was used for follow-up from 1991 to 1993, smoking status reported on the 1993 questionnaire was used for follow-up from 1993 to 1995, etc. Models for multivariate analyses for the NHSI included smoking status (never, past, or current <14, 15–24, or ≥25 cigarettes/d); physical activity (<1.0, 1.0–1.9, 2.0–3.9, 4.0–6.9, ≥7.0 h/wk), alcohol intake (0, 0.1–4.9, 5.0–9.9, ≥10 g/d); use of hormone replacement therapy (premenopausal, never, current, past); oral contraceptive use (ever or never); history of type 2 diabetes in parents or siblings (yes or no); consumption of coffee (0, 0.1–0.9, 1.0–1.9, 2.0–3.9, ≥4.0 cups/d), sugar-sweetened soft drinks (<1.0, 1.0–2.9, 3.0–6.9, ≥ 7cans/wk), fruit punch (nonalcoholic) (<1.0, 1.0–2.9, 3.0–6.9, ≥7 cans/wk); and quintiles of total energy intake, processed meat consumption, and the polyunsaturated-to-saturated fat intake ratio. Because of the different age range and questions on physical activity, models for multivariate analyses for the NHSII included the same variables with slightly different categories for smoking status (never, past, or current), physical activity (quintiles of metabolic equivalent h/wk), use of hormone replacement therapy (ever or never), oral contraceptive use (never, past, or current). There were no missing values for the dietary variables because only persons with valid dietary information were included.

The response to each biennial questionnaire exceeded 90% [24] and the number of missing values was low. In addition, the multiple repeated assessments allowed us to impute the most recent available data for missing values. For the remaining missing values, dichotomous indicator variables were included in the multivariate model. To test for linear trends across quintiles of intake, the quintile medians were modeled as a continuous variable. Modeling of multiplicative interaction terms for age and whole grain intake did not suggest that the proportional hazards assumption was violated (NHSI: *p* = 0.42, NHSII: *p* = 0.87 for the multivariate model). Pearson correlations were calculated between dietary intakes with adjustment for total energy intake. The proportion of the association between whole grain intake and risk of type 2 diabetes explained by BMI and the corresponding 95% confidence interval (CI) was estimated as described by Lin et al. based on the change in regression coefficients after adding BMI to the multivariate model [25]. *p*-Values were two tailed, and values less than 0.05 were considered statistically significant. The SAS statistical program version 9.1 (SAS Institute, http://www.sas.com/software/) was used for the analyses.

### Meta-analysis

**Figure 1:**
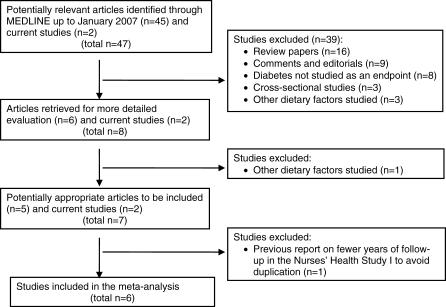
Flow Diagram of the Selection of Studies for the Meta-Analysis

The MEDLINE and EMBASE database was searched up to January 2007 for published articles on cohort studies that examined whole grain intake in relation to risk of type 2 diabetes. Our criteria for including studies in our meta-analysis were: prospective cohort study, type 2 diabetes as the endpoint, description of the whole grain assessment, presentation of RR with a measure of variability, and description of adjustment for potential confounders. Keywords used to identify relevant articles were: “diabetes mellitus, type 2” (as standardized medical subject heading [MeSH] term) AND (“whole grains” OR “whole grain”). Our MEDLINE search of English-language articles identified 45 abstracts of which six described potentially eligible studies. In addition, three non-English papers were identified that were all review articles. Full text review of the articles resulted in five cohort studies that met our criteria (Figure 1). One of these was NHSI [5], for which we included the updated analyses with longer follow-up. The search in EMBASE did not identify additional eligible studies. Broadening our search with: “diabetes mellitus, type 2” AND (“dietary fiber” OR “cereals”), all as MeSH terms, resulted in 356 items, but did not result in any additional eligible studies either. Together with the current study, a total of six studies were included in our meta-analysis.

Data extraction was independently performed by two of the authors (JSLdM, RMD) and there were no differences in extracted information. For each study, the RR of type 2 diabetes was expressed per two serving per day increment of whole grain intake, defining one serving as 30 g of grain for the study by Montonen et al. [8] and 20 g of whole grains for the current study. For NHSI, NHSII, and the Black Women's Health Study [9], we calculated the continuous estimate for a two-serving-per-day increment in whole grain intake. For the other three studies, we used the Greenland and Longnecker method to calculate a single continuous estimate and its estimated variance from the published information for quintiles or quartiles [26].

We used the STATA version 9.2 statistical program (STATA, http://www.stata.com/) for the meta-analysis. Summary measures were calculated from the logarithm of the RRs and corresponding standard errors of the individual studies using random effects models that incorporate both a within-study and an additive between-studies component of variance [27]. *p*-Values for heterogeneity of study results were calculated using the Cochran Q test [28]. Because this test depends on the number of studies and has limited sensitivity, we also expressed the degree of heterogeneity as the *I* ^2^ statistic [28]. The *I* ^2^ represents the percentage of total variation across studies that is due to between-study heterogeneity rather than chance. We observed that the between-studies heterogeneity in the standard meta-analysis could be due to the level of whole grain intake in the study population. To investigate this possibility, we conducted a meta-regression of log(RR) of the studies as the dependent variable on the log(median) whole grain intake of the study population [29]. We used the natural logarithm transformation of the median intake, because this fit the data better than the untransformed median intake and produced a plausible shape of the association. Begg and Egger tests and visual inspection of the funnel plot were used to evaluate possible publication bias [30,31].

## Results

### Nurses' Health Study I and II

We documented 4,747 cases of type 2 diabetes during 1,235,403 person-years of follow-up in the NHSI and 1,739 cases during 1,040,136 person-years in NHSII. Table 1 describes the characteristics of the study population according to whole grain consumption. Higher intakes of whole grain were associated with higher physical activity, a lower BMI, a lower likelihood of smoking, and a lower consumption of alcohol, soft drinks, and processed meats. Correlations with whole grain intake for NHSI and NHSII respectively were 0.75 and 0.75 for bran, 0.57 and 0.66 for germ, 0.79 and 0.77 for cereal fiber, and 0.53 and 0.53 for magnesium.

**Table 1:**
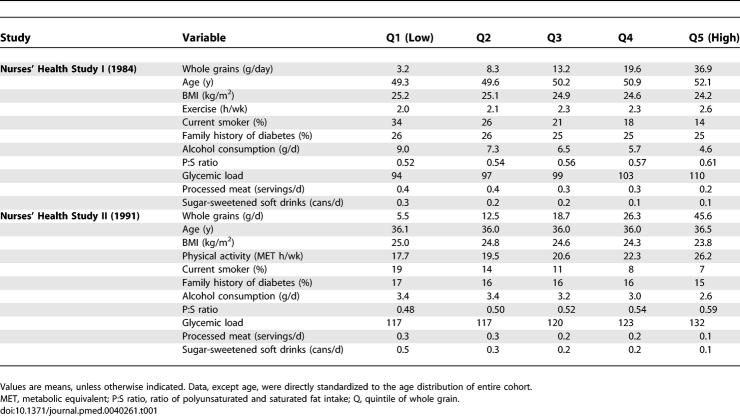
Baseline Characteristics of the Study Population by Whole Grain Consumption

Whole grain intake was inversely associated with risk of type 2 diabetes after adjustment for age and after adjustment for other potential confounders in both NHSI and NHSII (Table 2). Further adjustment for BMI, which may partly mediate the association with type 2 diabetes, substantially weakened the association, but significant inverse associations remained (Table 2). Further adjustment for magnesium intake did not substantially explain the inverse association for whole grain intake in either NHSI (RR 0.76, 95% CI 0.68–0.85 for extreme quintiles, *p*-value test for trend <0.001) or NHSII (RR 0.82, 95% CI 0.68–0.99, *p*-value 0.02). In the multivariate analysis, each 40 g increment in whole grain intake was associated with a RR of diabetes of 0.54 (95% CI 0.48–0.61) for NHSI and 0.64 (95% CI 0.54–0.76) for NHSII. After additional adjustment for BMI these RRs were 0.70 (95% CI 0.62–0.79) for NHSI and 0.83 (95% CI 0.70–0.98) for NHSII. BMI explained 42% (95% CI 33%–50%) of the association in NHSI and 57% (95% CI 29%–76%) of the association in NHSII.

**Table 2:**
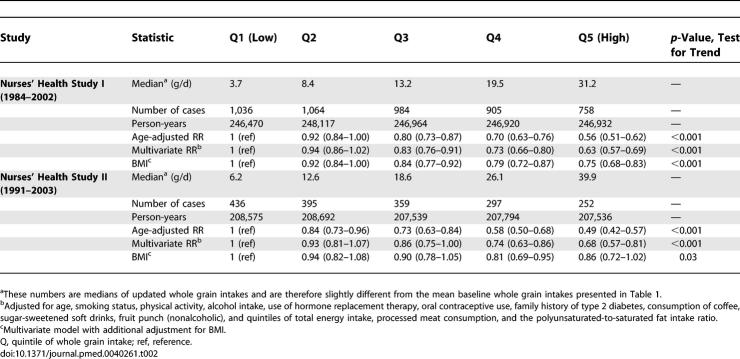
RR (95% CI) of Type 2 Diabetes According to Whole Grain Intake

Table 3 shows the results for bran and germ intake in relation to risk of type 2 diabetes. Associations for bran intake were similar to those for total whole grain intake, whereas associations with diabetes risk were weaker for germ intake. The correlation between bran and germ intake was 0.30 for NHSI and 0.37 for NHSII. Because associations in the fully adjusted model were significant for both bran and germ intake, we modeled bran and germ intake simultaneously for NHSI. After mutual adjustment, bran intake was significantly associated with a lower risk of type 2 diabetes (RR 0.70; 95% CI 0.62–0.79 for extreme quintiles; *p-*value, test for trend <0.001), whereas germ intake was not (RR 1.01; 95% CI 0.90–1.14; *p-*value, test for trend 0.91).

**Table 3:**
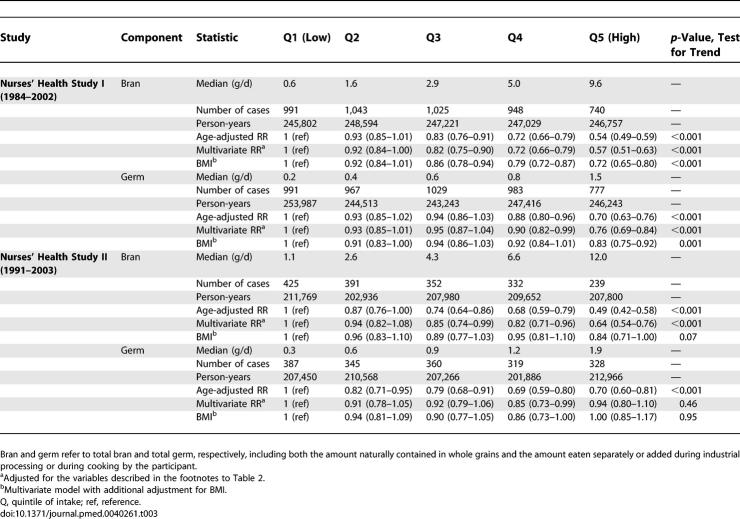
RR (95% CI) of Type 2 Diabetes According to Bran and Germ Intake

### Meta-Analysis

**Figure 2:**
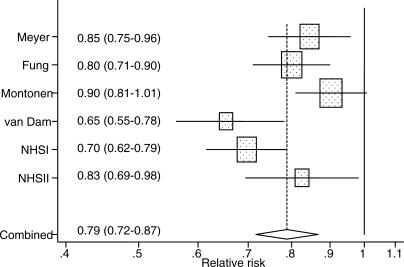
Forest Plot Showing the Multivariate-Adjusted RR of Type 2 Diabetes for a Two-Servings-per-Day Increment in Whole Grain Intake for Individual Cohort Studies and All Studies Combined Bars and the diamond indicate 95% CIs. The size of the squares corresponds to the weight of the study in the meta-analysis.

Characteristics of the six prospective cohort studies included in the meta-analysis are shown in Table 4. The cohorts included men and women, predominantly white or black populations, and participants from the United States and Finland. In addition to our cohorts, Cox proportional hazards analysis was used in three studies, but it was not reported whether the proportional hazards assumption was met [6,8,9]. Pooled logistic regression analysis was used in the other study [7]. Based on data from all studies combined, including 286,125 participants and 10,944 cases of type 2 diabetes, the pooled RR was 0.79 (95% CI 0.72–0.87) for each two-serving-per-day increment in whole grain intake (Figure 2). Although all studies were consistent with a substantial inverse association, there was significant heterogeneity in results (*I* ^2^ 68%, 95% CI 23%–86%; *p-*value, test for homogeneity 0.009). In the meta-regression analysis, a higher median whole grain intake of a study population (logarithmically transformed) was significantly associated with a weaker inverse association between whole grain intake and risk of type 2 diabetes (*p*-value, 0.03). The original heterogeneity was explained by this association: after median whole grain intakes of the population were accounted for, little heterogeneity in studies results remained (*I* ^2^ 5%, 95% CI 0%–80%; *p-*value, test for homogeneity 0.38). Similarly, after excluding the two studies that had a substantially lower [9] or higher [8] median whole grain intakes than the other studies (Table 4), the test for homogeneity was not significant anymore (*p-*value 0.15), while the pooled RR did not change (0.79; 95% CI 0.72–0.86). We also conducted a sensitivity analysis excluding one study at the time and calculating the pooled estimate for the remaining studies. The pooled RRs ranged from 0.76 (95% CI 0.70–0.84) after excluding the Finnish study [8] to 0.81 (95% CI 0.74–0.89) after excluding the Black Women's Health Study [9], indicating that the overall results were not unduly influenced by any one study. Visual inspection of the funnel plot (unpublished data) and the Begg (*p-*value 0.35) and Egger (*p-*value 0.30) tests did not suggest publication bias.

**Table 4:**
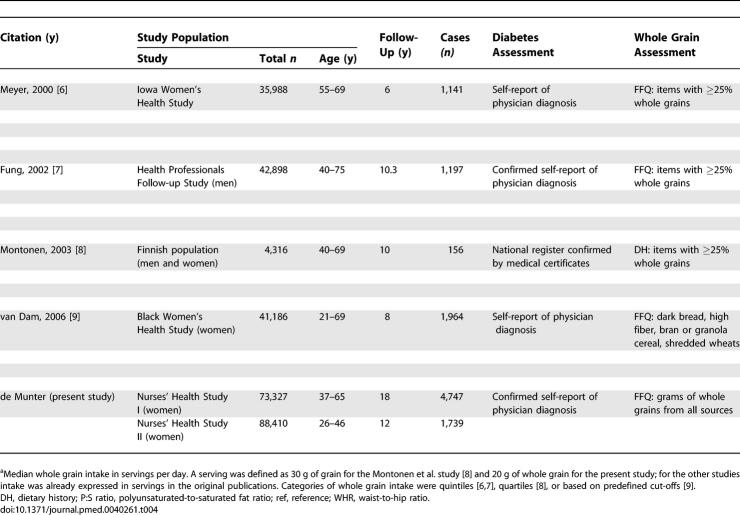
Cohort Studies of Whole Grain Consumption and Risk of Type 2 Diabetes

**Table 4:**
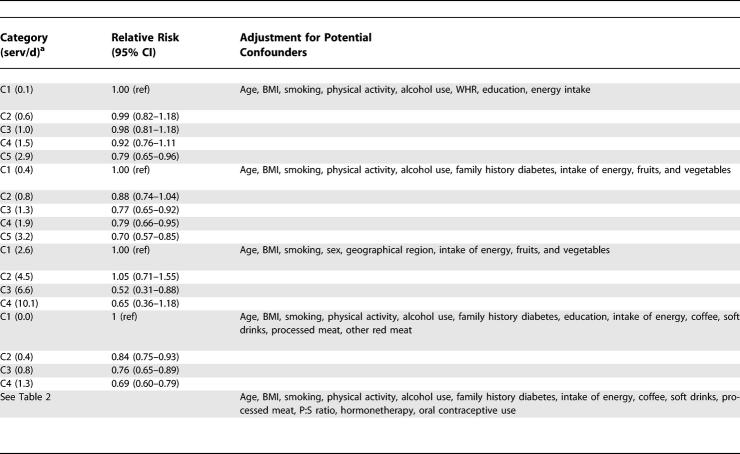
Extended.

## Discussion

In our prospective studies in over 150,000 women in their 20s through 60s at baseline, we observed a substantial inverse association between whole grain intake and risk of type 2 diabetes. Associations for total whole grain and bran intake were stronger than for germ intake, and we did not observe an independent association for germ intake after adjustment for bran intake. Based on a meta-analysis of six cohort studies, a two-servings-per-day increment in whole grain intake was associated with a 21% decrease in risk of type 2 diabetes.

### Strengths and Limitations

Strengths of our study included the prospective design and high rates of follow-up, which minimize the probability of recall bias or selection bias. Our study and the other studies included in the meta-analysis also had several potential limitations. First, although potential confounding was considered in detail, residual confounding by additional unmeasured or imperfectly measured confounders cannot be excluded. Particularly, higher whole grain intake tends to be associated with a healthier lifestyle, and incomplete adjustment for lifestyle factors could have led to overestimation of the strength of the inverse associations between whole grain intake and risk of type 2 diabetes. However, the consistency of findings across different cohorts and studies of different designs (see below) reduces the likelihood that residual confounding can fully explain the findings. Second, some measurement error in the assessment of dietary intakes is inevitable. Because of the prospective study design, misclassification of whole grain intake was unlikely to differ by case status and probably weakened the observed inverse association between whole grain intake and diabetes risk. We used averages of multiple repeated measurements of dietary intakes to reduce measurement error and better represent long-term diet [23]. Third, diabetes was assessed by self-report confirmed by a supplementary questionnaire, because screening for blood glucose was not feasible given the size of the cohorts. Data from our validation study using medical records suggest that reporting of diabetes is accurate for this population of registered nurses. Although underdiagnosis of diabetes is likely, it was probably limited in this cohort with ready access to medical care.

In the meta-analysis, the assessment of whole grain intake and its classification varied between the different included cohorts (Table 4). The use of an FFQ with less-detailed questions on whole grain foods [9] and the use of a classification that weighted all foods with at least 25% of whole grains equally [6–8] may have contributed to measurement error. In addition, the level of whole grain intakes differed substantially for the different cohorts. For example, the intake of whole grains and rye bread in particular was substantially higher for the Finnish population than for the US populations. Our results suggest that the benefit of adding a serving of whole grains may be greater for populations with a low intake than for those who already have a high intake, but this finding requires further research. Given the measurement error in the assessment of whole grain intake, the potential for residual confounding, and the difference in characteristics of the study populations, the estimated magnitude of associations should be interpreted with caution. However, all cohort studies were consistent with a substantial protective effect of whole grain consumption in relation to type 2 diabetes and excluding any one study did not substantially change the pooled estimate. Publication bias can affect the findings of any meta-analysis, but standard tests did not indicate the presence of publication bias in the current analysis.

### Relation to Other Studies

The findings from cohort studies are consistent with the direct association between whole grain consumption and insulin sensitivity that has been observed in cross-sectional studies in adolescent [32] and adult US populations [33–36]. Higher whole grain consumption was also associated with lower fasting and postload plasma glucose concentrations in one cross-sectional study [37], but not in two other studies [32,33]. In population of adults in Iran, whole grain consumption was inversely associated with newly detected abnormal glucose metabolism and type 2 diabetes [38]. Although all studies that quantified total whole grain consumption were included in our meta-analysis, two studies evaluated whole grain bread consumption in relation to type 2 diabetes. Whole grain bread intake was associated with a significantly lower risk of type 2 diabetes in a German cohort (RR 0.78, 95% CI 0.62–0.97 for ≥80 versus <4 g/d) [39], but not in an Australian cohort (RR 0.86, 95% CI 0.63–1.18 for highest versus lowest quartile) possibly due to the low reproducibility of the assessment of bread consumption in that cohort [40].

In a randomized cross-over trial in hyperinsulinemic overweight adults, consumption of whole grains (mostly whole wheat, rolled oats, and brown rice) for 6 wk increased insulin sensitivity as compared with refined grains [41]. Results of intervention studies of wheat bran have been mixed, with beneficial effects on glucose tolerance in studies in persons with [42] and without glucose intolerance [43], but no improvement in glycemic control in individuals with established type 2 diabetes [44]. In a randomized cross-over trial in postmenopausal women, consumption of high-fiber rye bread for 8 wk did not alter insulin sensitivity as compared with white wheat bread, but enhanced acute insulin response [45].

### Mechanisms

Adjustment for BMI substantially weakened the observed association between whole grain intake and risk of type 2 diabetes in our study, suggesting that a relation between whole grain intake and diabetes risk may be partly mediated by effects on body weight. Higher whole grain intake was associated with reduced weight gain in several cohort studies [15,46], but data from randomized trials are currently lacking.

The definition of whole grains used in the cohort studies did not require an intact kernel. Given the commercial availability of grains in the US, whole grain intake in the cohorts probably largely consisted of “shredded whole grains” such as whole wheat bread, which have glycemic indices that are similar to refined grains such as white bread [47]. Therefore, a low dietary glycemic index or glycemic load seems an unlikely explanation for the observed inverse association between whole grain intake and diabetes risk.

Whole grains are an important source of cereal fiber, vitamins, minerals, lignans, and other phytochemicals [10]. Magnesium intake improved glucose metabolism in some short-term clinical trials [12] and was inversely associated with risk of type 2 diabetes in several cohort studies [9,39]. However, magnesium intake did not explain the inverse association between whole grain intake and risk of type 2 diabetes in the current study. Higher cereal fiber intake has generally been associated with a lower risk of type 2 diabetes in cohort studies [39]. Furthermore, intake of purified insoluble cereal fiber intake for 3 d increased insulin sensitivity in a randomized cross-over study [11]. In a shorter trial, intake of these fibers stimulated the acute secretion of glucose-dependent insulinotropic polypeptide and insulin and reduced the glucose response to a meal the following day [48]. Intake of lignans reduced the development of diabetes mellitus in animal studies, possibly through their antioxidant or (anti) estrogenic effects [13]. Further mechanistic studies are needed to elucidate effects of whole grain constituents or combinations on glucose metabolism.

### Conclusions

Findings from prospective cohort studies consistently indicate that higher consumption of whole grains can contribute to the prevention of type 2 diabetes. Cross-sectional studies and short-term randomized trials have provided additional evidence for beneficial effects of whole grains on glucose homeostasis. Taken together, evidence for beneficial metabolic effects is stronger for consuming a variety of whole grains than for wheat bran in isolation. These data provide further support for recommendations to increase consumption of whole grains including whole wheat, whole oats, oatmeal, whole grain corn and popcorn, brown and wild rice, whole rye, whole grain barley, buckwheat, triticale, bulgur, millet, quinoa, and sorghum [49]. The US Department of Agriculture defines one serving of whole grains as 16 g of whole grain ingredients, the equivalent of the content of a one-ounce (28.4 g) slice of 100% whole wheat bread, but expressing whole grain intakes and the whole grain content of foods directly in grams rather than servings may also be a useful method to communicate amounts of whole grains. Educational efforts and clear information on whole grain contents on food labels can contribute to the recognition of foods high in whole grains by consumers. The consumption of whole grains in many populations is very low, an average of one serving per day for US adults [50] and even less in British adults [51], suggesting that increased consumption has the potential to contribute substantially to reducing risk of type 2 diabetes in these populations.

## Abbreviations

BMI: body mass index
CI: confidence interval
FFQ: food frequency questionnaire
NHS: Nurses' Health Study
RR: relative risk
